# Stannic Oxide Nanoparticle Regulates Proliferation, Invasion, Apoptosis, and Oxidative Stress of Oral Cancer Cells

**DOI:** 10.3389/fbioe.2020.00768

**Published:** 2020-07-17

**Authors:** Hui Li, Qiushi Li, Yingcai Li, Xue Sang, Haotian Yuan, Baihong Zheng

**Affiliations:** ^1^Department of Stomatology, China-Japan Union Hospital, Jilin University, Changchun, China; ^2^VIP Integrated Department, School and Hospital of Stomatology, Jilin University, Changchun, China; ^3^Department of Pediatrics, The Second Hospital of Jilin University, Changchun, China

**Keywords:** SnO_2_ nanoparticles, oral cancer, proliferation, invasion, apoptosis, oxidative stress

## Abstract

**Objective:**

To explore the effects of SnO_2_ nanoparticles (NPs) on proliferation, invasion, apoptosis, and oxidative stress of oral cancer.

**Methods:**

SnO_2_ NPs were prepared and characterized. Oral cancer cell lines CAL-27 and SCC-9 were cultured *in vitro*. We detected the effects of various concentrations of SnO_2_ NPs (0, 5, 25, 50, 100, 200 μg/mL) on the proliferation of oral cancer cells, and observed the morphological changes, and measured the cells ability of migration, invasion and apoptosis condition, and the levels of oxidative stress were measured by detecting malondialdehyde (MDA) and reactive oxygen species (ROS). Besides, we also measured the changes of mRNA and protein levels of factors related to cell proliferation, migration, invasion, apoptosis, and oxidative stress.

**Results:**

SnO_2_ NPs inhibited the proliferation of oral cancer cells in a concentration-dependent manner (all *P* < 0.05). And SnO_2_ NPs treatment could reduce the migration and invasion ability of cells (all *P* < 0.05), induce apoptosis, and those effects were better when treated for 48 h than 24 h (all *P* < 0.05). And SnO_2_ NPs could induce oxidative stress in cells (all *P* < 0.05). Besides, the concentrations of cyclin-D1, C-myc, matrix MMP-9, and MMP-2 in SnO_2_ NPs treated group was decreased (all *P* < 0.05), and the expression levels of cleaved Caspase-3, cleaved Caspase-9, and Cytochrome C were increased (all *P* < 0.05).

**Conclusion:**

In the present study, we found that SnO_2_ NPs could play a cytotoxic role in oral cancer cells, and inhibit cell proliferation, migration, and invasion, and induce oxidative stress and apoptosis, which suggests that SnO_2_ NPs may have the effects of anti-oral cancer. However, a more in-depth study is needed to determine its roles.

## Introduction

Oral cancer is one of the most common human malignant tumors in the world (Shiga et al., 2011; Omori et al., 2020). Despite progress in cancer treatment, for patients with oral cancer, the 5-year survival rate is still less than 50% (Cristaldi et al., 2019). At present, the available treatments for oral cancer mainly include surgery, radiotherapy, and chemotherapy. After surgical treatment, it is easy to relapse, and the prognosis is poor. Although chemotherapy has effects to some extent, the tolerance and drug resistance of chemotherapy drugs are still severe problems faced by oral cancer patients. Therefore, it is necessary to find and develop a more effective and safer chemical molecular drug for treating the disease.

With the continuous development of nanomedicines, nanoparticles are now considered as a potential cancer treatment with bright future (Ding et al., 2019; Feng et al., 2019; Jiang et al., 2019; Li et al., 2019; Qi et al., 2019; Sun et al., 2019; Wang et al., 2019; Xu et al., 2019; Xiong et al., 2020; Yang et al., 2020). The nanoparticle is small and can facilitate the various biological reactions (Behzadi et al., 2017; Chen et al., 2017a). Tin oxide (SnO_2_) is an essential n-type wide bandgap (3.6 eV) semiconductor material. Currently, SnO_2_ nanoparticles are being used in different fields, including solar cells, gas sensors, catalysts, lithium-ion batteries, solid-state chemistry, etc. (Cheng et al., 2014). Increasingly, SnO_2_ NP has more and more extensive use in the field of biomedicine, and studies have shown that SnO_2_ NP has antibacterial and antioxidant activity (Vidhu and Philip, 2015). For examples, in cell-related research, Tammina et al. (2017) observed that SnO_2_ NP can exert anti-human colorectal cancer and anti-lung cancer effects through cytotoxicity; Roopan et al. (2015) reported the cytotoxic response of SnO_2_ NP to hepatocellular carcinoma (Roopan et al., 2015); Ahamed et al. (2018) demonstrated that SnO_2_ NP could induce breast cancer cell toxicity through oxidative stress. In animal-related research, Yang et al. (2017) found that SnO_2_ NP had an inhibitory effect on the weight gain of newborn rats. However, there is still little research on the mechanism of toxic effects of SnO_2_ NP at the cellular and molecular levels. Therefore, before SnO_2_ NP can be applied in the field of biomedicine, in-depth research on the possible biological roles of SnO_2_ is essential (Dong et al., 2012).

Since there is no relevant research on the role of SnO_2_ NP in the initialization and progression of oral cancer, two oral cancer cells CAL-27 and SCC-9 were selected in this study to explore the possibility of SnO_2_ NP in treating oral cancer by observing its effect on proliferation, invasion, apoptosis, and oxidative stress of the cells.

## Materials and Methods

### Synthesis of SnO_2_ NP

SnO_2_ NP was synthesized by the sol-gel method, see reference for details (Aziz et al., 2013). First, 5.0 g of SnCl_4_⋅5H_2_O was dissolved in 200.0 mL of ethanol (C_2_H_5_OH) and stirred for 30 min by a magnetic stirrer. Then, 6.0 mL of acetylacetone was added to hydrolyze SnO_2_, and the solution was refluxed through an 80°C condenser. After that, the solution was further dried for 3 h in a 90°C hot air oven (Thermo Fisher Scientific, United States) to obtain SnO_2_ gel, which was then calcined at 400-500°C for 2 h to obtain SnO_2_ NP.

### Characterizations of SnO_2_ NP

The absorption spectrum of SnO_2_ NP was measured using a spectrometer (UV-1800, Shimadzu, Japan) at a resolution of 0.5 nm and a wavelength range of 300–900 nm. The structure of SnO_2_ NP was analyzed by using Ku-Cα ray powder X-ray diffraction (XRD) with a wavelength of 1.54056 Å, and the XRD spectrum in the surrounding environment was recorded at a scan rate of 0.02°/s in the 2θ range of 20–80°. Besides, the characteristics of SnO_2_ NP were observed with a transmission electron microscope (TEM).

### Cell Culture

Human oral cancer cell lines CAL-27 and SCC-9 were purchased from ATCC (United States). The cells were incubated in DMEM medium containing 10% fetal bovine serum (Gibco, United States), 100 U/mL penicillin, and 100 μg/mL streptomycin (Invitrogen, United States), with 5% carbon dioxide (CO_2_) at 37°C.

### Experimental Grouping and Treatment of Cells Exposed to SnO_2_ NP

DMEM was used to prepare a SnO_2_ NP storage solution (1.0 mg/mL), which was then diluted to an appropriate concentration (5–200 μg/mL). Before the experiment, SnO_2_ NP of different concentrations was placed in the ultrasonic bath with 40 W for 15 min at room temperature, which could maintain nanoparticles being evenly distributed in DMEM and prevent aggregation. The cells were divided into the control group (DMEM group, 0 μg/mL) and experimental groups (SnO_2_ NP concentration was 5, 25, 50, 100, and 200 μg/mL), and two experimental treatment time points of 24 and 48 h were set.

### Detection of Cell Proliferation Behaviors

Cell Counting Kit-8 (CCK-8, Donjindo, Japan) was used for the measurement. CAL-27 and SCC-9 cells were inoculated into 96-well plates at a density of 1 × 10^4^ cells/well. After 24 or 48 h of treatment for control and experimental groups, 10 μL of CCK-8 solution was added to each well for another 1 h of incubation at 37°C. After that, the absorbance at 450 nm (Absorbance, A) was measured with a multifunctional microplate reader (Bio-Rad, Untied States). A blank well (only containing medium and CCK-8 solution) was set in the experiment to counteract background interference. The formula for calculating cell proliferation activity is as follows (Chen et al., 2017b; Li et al., 2018):

(1) $$\mathrm{Cell}\mathrm{Viability}\left(\%\right)=\left(A_{\mathrm{experimental}\,\mathrm{group}}-A_{\mathrm{blank}\,\mathrm{group}}\right)/\left(A_{\mathrm{control}\,\mathrm{group}}-A_{\mathrm{blank}\,\mathrm{group}}\right)\times 100\%.$$

Each group has four replicates.

### Cell Morphology Observation

The morphological changes of cells in treatment groups were determined by a phase-contrast inverted microscope (Leica Microsystems Inc., United States).

### Cell Scratch Test

First, cells were prepared for the control and experimental groups respectively, and then CAL-27 and SCC-9 cells were seeded on 6-well plates at a density of 2 × 10^5^ cell/well and cultured for 24 h. Straight lines parallel to the cell layer were then drawn using a sterile pipette tip. After that, the control and experimental groups were treated accordingly, and the cells were photographed at 0 and 24 h with the use of a phase-contrast inverted microscope. Finally, ImageJ was used to measure the width of the scratch area and calculate the cell migration rate. The formula is as follows: cell migration rate = (scratch width at 0 h−scratch width at 24 h) / scratch width at 0 h × 100%.

### Cell Invasion Experiment

Transwell was used for the cell invasion test (Chen et al., 2017b). First, cells were prepared for the control group and the experimental group. Then CAL-27 (6 × 10^4^ cell/well) and SCC-9 cells (8 × 10^4^ cell/well) inoculated into 200 μL of serum-free medium were plated in the upper chamber coated with Matrigel, and 500 μL of medium containing 10% fetal calf serum was added in the lower chamber. The cells were incubated at 37°C for 24 h, after which the cells on the membrane surface were fixed with 4% paraformaldehyde, stained with 0.1% crystal violet, and placed in a phase-contrast inverted microscope for cell counting.

### Detection of Oxidative Stress Indicators

The oxidative stress was evaluated by determining the levels of malondialdehyde (MDA) and reactive oxygen species (ROS). According to the instructions, the MDA detection kit for lipid peroxidation (S0131, Biyuntian, China) and the active oxygen detection kit (S0033, Biyuntian, China) were used for detection, respectively.

### Western Blot to Detect Protein Expression

The cells after the experimental treatment were washed with pre-cooled PBS and lysed with a strong RIPA lysis buffer (Biyuntian, China) containing protease inhibitors, which was subsequently centrifuged at 16,000 *g* at 4°C for 20 min to retain the supernatant. Then, the protein quantification was measured by the BCA protein concentration detection kit (Biyuntian, China). After that, the protein was denatured by heating at 98°C for 10 min, and separated by SDS-PAGE gel electrophoresis. After electrophoresis, proteins on the gel were transferred to PVDF membrane (Millipore, United States), and the membrane was then blocked with a blocking solution (Biyuntian, China) for 1 h after the transfer. Subsequently, the membrane was incubated overnight at 4°C after the addition of the corresponding primary antibody. On the next day, the membrane was washed with TBST three times, and the secondary antibody conjugated with the corresponding horseradish peroxidase (HRP) was then incubated at room temperature for 2 h. After that, the membrane was washed three times with TBST, and the Western blot was developed with ECL color developing solution (Biyuntian, China). Finally, the grayscale analysis was performed with Photoshop CS6. The primary antibodies used in this experiment were cleaved Caspase-3 (ab2302 17 kDa), Caspase-3 (ab13847 17 kDa), cleaved Caspase-9 (ab2324 46 kDa), Caspase-9 (ab202068 46 kDa), matrix metalloproteinase 9 (Matrix metalloproteinase-9, MMP-9) (ab38898 92 kDa), MMP-2 (ab97779 74 kDa), G1/S-specific cyclin-D1 (Cyclin D1, CCND1) (ab134175 34 kDa), c-myc (Ab32072 57 kDa), Cytochrome C (ab133504 14 kDa), and β-actin (ab227387 42 kDa), of which β-actin serves as an internal reference protein.

### Flow Cytometry to Detect Apoptosis

The flow cytometer Annexin V-FITC/PI double staining method was used for the detection. CAL-27 and SCC-9 cells were seeded on 6-well plates at a density of 2 × 10^5^ cells/well. After treatment for 24 or 48 h in the control and experimental groups, cells were digested with trypsin digestion solution without EDTA and centrifuged at 1,000 rpm for 5 min at room temperature to retain the cell pellet. Then, the cells washed with 1 mL of pre-chilled PBS were centrifuged at 3,000 rpm for 5 min at room temperature to retain the cell pellet, which was followed by PBS washing twice. After that, the cell pellet was added with 500 μL of binding buffer to resuspend, 10 μL PI and 5 μL Annexin V-FITC were added, and cultured in the dark. After incubation, the apoptosis was immediately analyzed using flow cytometry (BD Biosciences, United States).

### Real-Time Fluorescence Quantitative PCR to Detect Expression Level of Target Genes

The cells after the experimental treatment were washed with pre-cooled PBS, and the total RNA was extracted from the cell line using TRIzol^®^ reagent (Invitrogen, United States). After that, the concentration and purity of the RNA were measured with a multifunctional microplate reader. According to the instructions of the reverse transcription kit (Takara, Japan), 1 μg of total RNA was used for PCR to obtain cDNA. Then, the SYBR Green kit (Takara, Japan) and target gene primers or internal reference gene (β-actin) primers were used to perform real-time fluorescence quantitative PCR (RT-qPCR). Finally, the expression cycle Ct value of each gene was measured, and the relative expression level was calculated according to this formula 2^–ΔΔ*Ct*^. Primer sequences are shown in Table 1.

**TABLE 1:** Primer sequence.

**Gene**	**Forward (5′→3′)**	**Reverse (5′→3′)**
MMP-9	CGCCAGTCCACCCTTGTG	CAGCTGCCTGTCGGTGAGA
MMP-2	CGTCTGTCCCAGGATGACATC	ATGTCAGGAGAGGCCCCATA
CCND1	GAACTACCTGGACCGCTTCC	TAGATGCACAGCTTCTCGGC
C-myc	AGCAAACCTCCTCACAGCCC	ACTGTCCAACTTGACCCTCT
Cytochrome C	CTTTGGGCGGAAGACAGGTC	TTATTGGCGGCTGTGTAAGAG
β-actin	CATGTACGTTGCTATCCAGGC	CTCCTTAATGTCACGCACGAT

## Statistical Analysis

All experiments were independently repeated three times. The measurement data obtained in the experiment are expressed as mean ± standard deviation, and software SPSS 18.0 was used for statistical analysis. Student’s *t*-test was used for comparison between the two groups, and one-way ANOVA was used for comparison within the group when considering the single factors. *P* < 0.05 was considered statistically significant.

## Results

### Physicochemical Characterization of SnO_2_ NP

**FIGURE 1:**
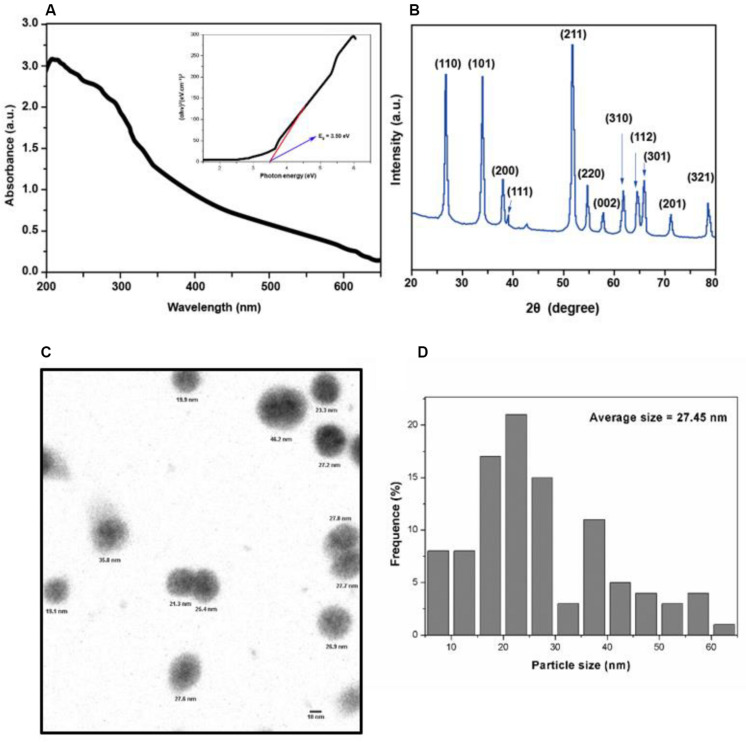
Characterization of SnO_2_ NP. **(A)** Absorption spectra of SnO_2_ NP. Figure inset represents the bandgap energy of SnO_2_ NP. **(B)** XRD spectra of SnO_2_ NP. **(C,D)** TEM micrograph of SnO_2_ NP and a histogram of the size distribution of SnO_2_ NP.

As shown in Figure 1A, the absorption spectrum of SnO_2_ NP ranges from 200 to 700 nm. The formula calculates the absorption coefficient (α) of SnO_2_ NP: α = A/d (A: absorbance, d: cuvette thickness) (Khan et al., 2014). According to the formula: (αhν) = A (*hν*−Eg) (where hν is the photon energy, A is a constant that does not depend on the photon energy), the absorption coefficient α was used to make Tauc plots (Khan et al., 2017). The analysis found that the energy band gap of SnO_2_ NP is 3.50 eV, which is consistent with other reports (Gattu et al., 2015; Mohanta et al., 2017). It is also known that the energy band gap of semiconductor NP plays a critical role in the toxicity of tumor cells (Rasmussen et al., 2010).

X-ray diffraction analyzed the crystal structure of SnO2 NP. As shown in Figure 1B, all peaks in XRD are related to the rutile structure of SnO_2_ (JCPDS No. 41-1445). According to the Scherrer equation: *d* = Kλ / βCos θ (where *K* = 0.9 is the shape factor, λ is the X-ray wavelength of Cu Kα rays (1.54 Å), θ is the Bragg diffraction angle, and β is the diffraction line at its maximum intensity (broadness) measured at half a radian), it is found that the average size of SnO_2_ NP is about 13 nm, and the XRD results are consistent with the results reported by other studies (Chen et al., 2014).

The appearance of SnO_2_ NP was detected by TEM and shown in Figure 1C. The average TEM size of SnO_2_ NP was calculated based on more than 300 nanoparticles and found that the average TEM size was 27.45 ± 13.4 nm (Figure 1D).

### SnO_2_ NP Inhibit Proliferation of Oral Cancer Cells

**FIGURE 2:**
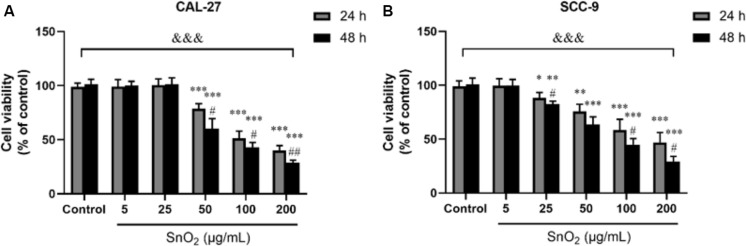
SnO_2_ NP inhibited cell viability in oral cancer cells. **(A,B)** The viability of CAL-27 and SCC-9 cells was measured after treatment of different concentrations of SnO_2_ NP at 24 or 48 h. *n* = 4, compared with control group, *P < 0.05, **P < 0.01, ***P < 0.001; compared with 24 h treated group, ^#^*P* < 0.05, ^##^*P* < 0.01; compared in groups, ^&⁣&⁣&^*P* < 0.001.

Figure 2A showed that compared with the control group, SnO_2_ NP induced a significant decrease in the proliferation activity of CAL-27 cells in a concentration-dependent manner (50, 100, and 200 μg/mL) (all *P* < 0.05), and the inhibition effect was more significant at 48 h than that at 24 h. When the concentration of SnO_2_ NP was lower than 50 μg/mL (5, 25 μg/mL), there was no significant effect on cell proliferation activity (all *P* > 0.05). Figure 2B showed that compared with the control group, SnO_2_ NP induced a significant decrease in the proliferation activity of SCC-9 cells in a concentration-dependent manner (25, 50, 100, and 200 μg/mL) (all *P* < 0.05), and the treatment at 48 h has stronger effects on cell proliferation activity than that at 24 h. When the concentration of SnO_2_ NP was less than 5 μg/mL, there was no significant effect on cell proliferation activity (*P* > 0.05). Since SnO_2_ can exert toxicity on oral cancer cells when the concentration greater than 50, 100 μg/mL was used as the treatment concentration in the SnO_2_ NP group.

### SnO_2_ NP Changed Density Morphology of Oral Cancer Cells

**FIGURE 3:**
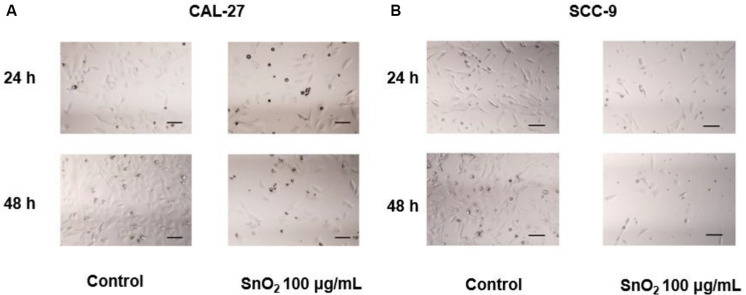
SnO_2_ NP alters cell density and morphology in oral cancer cells (100×, 100 μm). **(A,B)** The changes in cell density and morphology in CAL-27 and SCC-9 cells.

Figure 3A showed that compared with the control group, the treatment of 100 μg/mL SnO_2_ NP can significantly reduce the CAL-27 cell density, and the cell density was changed more significantly with the treatment of SnO_2_ NP at 48 h than that at 24 h. As shown in Figure 3B, the changes caused by SnO_2_ NP on SCC-9 cells are consistent with the results from CAL-27 cells.

### SnO_2_ NP Inhibited Migration and Invasion of Oral Cancer Cells

**FIGURE 4:**
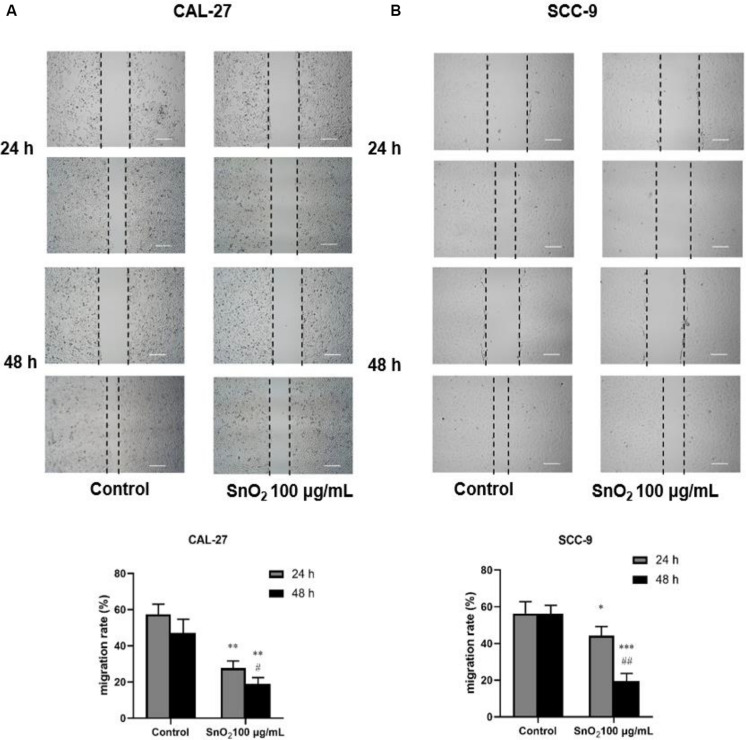
SnO_2_ NP inhibited cell migration in oral cancer cells (50×, 200 μm). **(A,B)** The ability of cell migration was measured in CAL-27 and SCC-9 cells after treatment of 0 or 100 μg/mL SnO_2_ NP at 24 or 48 h. Compared with control group, *P < 0.05, **P < 0.01, ***P < 0.001; compared with 24 h treated group, ^#^*P* < 0.05, ^##^*P* < 0.01.

As shown in Figures 4A,B, compared with the control group, SnO_2_ NP treatment significantly suppressed the migration of CAL-27 and SCC-9 cells (both *P* < 0.05), and cells treated with SnO_2_ NP for 48 h has a weaker migration ability than those treated for 24 h (all *P* < 0.05).

**FIGURE 5:**
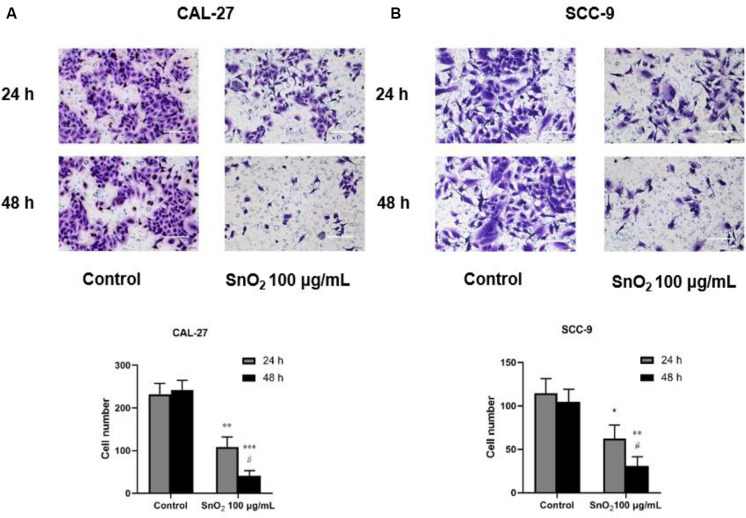
SnO_2_ NP inhibited cell invasion of oral cancer cells (100×, 100 μm). **(A,B)** The ability of cell invasion was measured in CAL-27 and SCC-9 cells after treatment of 0 or 100 μg/mL SnO_2_ NP at 24 or 48 h. Compared with control group, *P < 0.05, **P < 0.01, ***P < 0.001; compared with 24 h treated group, ^#^*P* < 0.05.

As shown in Figures 5A,B, compared with the control group, SnO_2_ NP treatment significantly reduced the invasive ability of CAL-27 and SCC-9 cells (both *P* < 0.05), and the effect on cell invasion was more significant with the treatment of SnO_2_ NP at 48 h than at 24 h (*P* < 0.05).

### SnO2 NP Induced Apoptosis of Oral Cancer Cells

**FIGURE 6:**
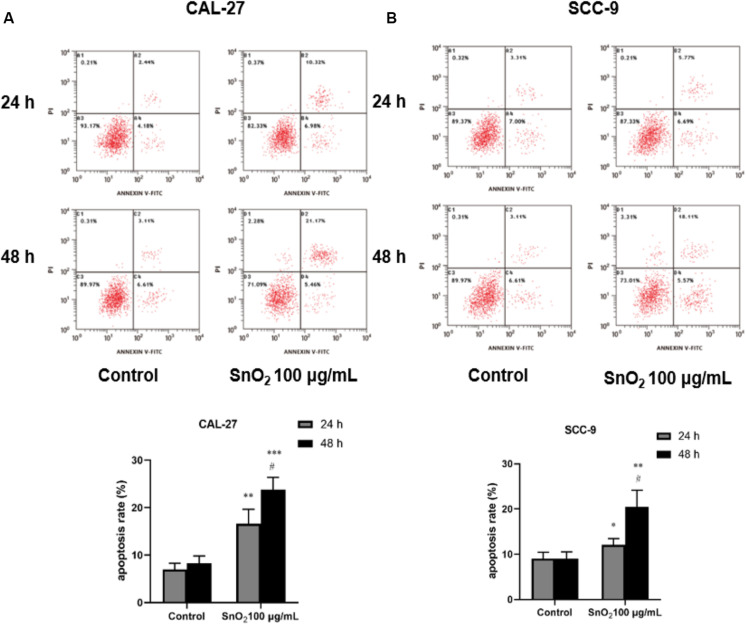
SnO_2_ NP induced cell apoptosis in oral cancer cells. **(A,B)** Apoptosis was determined in CAL-27 and SCC-9 cells after treatment of 0 or 100 μg/mL SnO_2_ NP at 24 or 48 h. Compared with control group, *P < 0.05, **P < 0.01, ***P < 0.001; compared with 24 h treated group, ^#^*P* < 0.05.

Figure 6A indicated that compared with the control group, 100 μg/mL SnO_2_ NP can significantly induce apoptosis of CAL-27 cells (*P* < 0.05), and the cells treated with SnO_2_ NP at 48 h have more significant change than that at 24 h. As shown in Figure 6B, the apoptosis induced by SnO_2_ NP on SCC-9 cells was consistent with results with CAL-27 cells (*P* < 0.05).

### SnO_2_ NP Induced Oxidative Stress in Oral Cancer Cells

**FIGURE 7:**
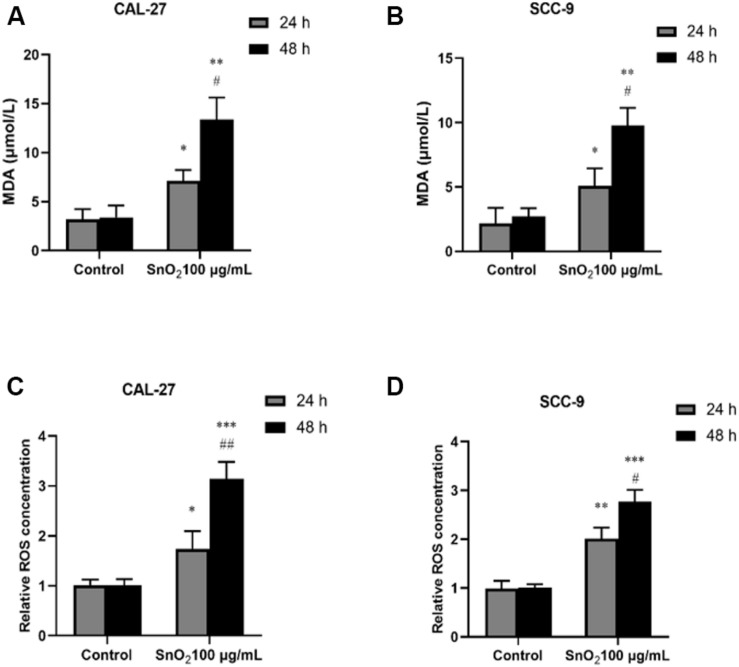
SnO_2_ NP induced cell apoptosis in oral cancer cells. **(A,B)** MDA levels were determined in CAL-27 and SCC-9 cells after treatment of 0 or 100 μg/mL SnO_2_ NP at 24 or 48 h. **(C,D)** The effects of 0 or 100 μg/mL SnO_2_ NP on ROS concentrations were tested in CAL-27 and SCC-9 cells at 24 or 48 h. Compared with control group, *P < 0.05, **P < 0.01, ***P < 0.001; compared with 24 h treated group, ^#^*P* < 0.05, ^##^*P* < 0.05.

As shown in Figures 7A,C, CAL-27 cells treated with 100 μg/mL SnO_2_ NP, compared with those of the control group, significantly increased the levels of oxidative stress-related factors MDA and ROS (both *P* < 0.05), and the cells treated with at 48 h changed more significant than at 24 h. As shown in Figures 7B,D, the SnO_2_ NP-induced oxidative stress of SCC-9 cells was consistent with results from CAL-27 cells (*P* < 0.05).

### SnO_2_ NP Altered mRNA and Protein Expression of Oral Cancer Cell Proliferation, Migration, Invasion, Apoptosis, and Oxidative Stress-Related Genes

CAL-27 cells are more sensitive to SnO_2_ NP stimulation in terms of migration, invasion, and apoptosis, and were selected in the study for the Western blot and RT-qPCR experiments.

**FIGURE 8:**
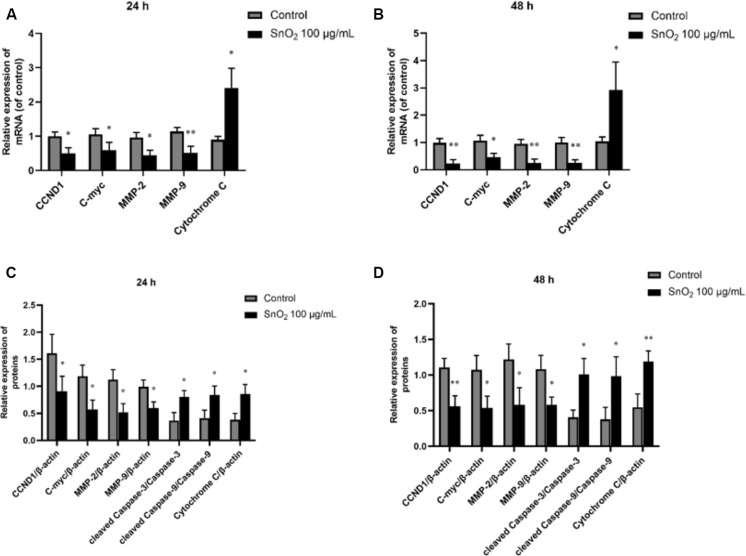
SnO_2_ NP changed expression of proliferation, migration, invasion, apoptosis, and oxidative stress-related protein. **(A,B)** The changes of target mRNA levels in cells after treatment of 0 or 100 μg/mL SnO_2_ NP at 24 or 48 h. **(C,D)** The changes of target protein levels in cells after treatment of 0 or 100 μg/mL SnO_2_ NP at 24 or 48 h. Compared with a control group, *P < 0.05, **P < 0.01.

As shown in Figures 8A,B, compared with the control group, 100 μg/mL SnO_2_ NP treatment at both 24 and 48 h could not only significantly inhibit the expression of proliferation-related factors CCND1 and c-myc in CAL-27 cells to varying degrees, but also decrease the mRNA levels of migration and invasion related factors MMP-2 and MMP-9 (both *P* < 0.05), to promote the mRNA expression of oxidative stress-related factors Cytochrome C (both *P* < 0.05).

Moreover, as shown in Figures 8C,D, compared with the control group, 100 μg/mL SnO_2_ NP treatment at 24 and 48 h can not only significantly inhibit the expression of proliferation-related factors CCND1, c-myc in CAL-27 cells, but also decrease the protein levels of migration and invasion related factors MMP-2 and MMP-9 (both *P* < 0.05), promote the expression of such apoptosis-related proteins as cleaved Caspase-3, cleaved Caspase-9 and oxidative stress-related factor Cytochrome C (all *P* < 0.05).

## Discussion

SnO_2_ is a multifunctional metal oxide. Since SnO_2_ NP can be used as antibacterial agents, disinfectants, and other products with excellent performance, SnO_2_ NP has been gradually applied in the medical field. Recently, the critical role of SnO_2_ NP in cancer treatment has attracted widespread attention. Some studies have confirmed that SnO_2_ NP had a robust anti-cancer effect on specific cancer cells. For instance, SnO_2_ NP could induce human HCT116 and A549 cells to produce ROS *in vitro*, which leads to cell death (Tammina et al., 2017); besides, it is found that SnO_2_ NP treatment can cause HepG2 cells to occur Morphological changes and cytotoxic effects (Roopan et al., 2015); it is also demonstrated that SnO_2_ NP can induce oxidative stress in MCF-7 cells, prompting the cells to synthesize ROS, increasing H_2_O_2_ levels, triggering lipid peroxidation, reducing glutathione and antioxidant enzymes levels, and finally causing cell damage (Ahamed et al., 2018); Moreover, Lv found that SnO_2_ nanofibers can not only reduce the cell activity of hepatocellular carcinoma SMMC-7721 but also induce apoptosis. Western blot detection found that the expression of Caspase-3, the apoptosis-related protein, was increased (Lv et al., 2018).

In this study, SnO_2_ NP was prepared by the sol-gel method. Spectral analysis, XRD analysis, and TEM observation confirmed that the SnO_2_ NP that we produced were consistent with literature records and could be used for experiments. This study first found that SnO_2_ NP had anti-proliferative effects on two different kinds of oral cancer cells. Specifically, SnO_2_ NP induced a decrease in the activity of oral cancer cells in a dose-dependent manner, which became more evident with longer exposure time. This result is consistent with previous studies on the effects of SnO_2_ NP on other cancers (Roopan et al., 2015; Tammina et al., 2017). At the same time, the protein and mRNA expressions of CCND1 and c-myc, the cell proliferation-related genes, were also detected. Some studies have found that CCND1 can regulate the cell cycle by controlling the G1/S phase transition, and increased expression of CCND1 will lead to the occurrence of cancer (Musgrove et al., 2011). As a carcinogenic transcription factor, C-Myc recognizes E-box and its related sequences in the target gene promoter. The increase of C-Myc level significantly improves the tumor proliferation ability (Liu et al., 2015). The study indicated that SnO_2_ NP could inhibit the expression of CCND1 and c-myc in oral cancer cells, thereby exerting an inhibitory effect on cell proliferation. Also, the morphological changes in oral cancer cells after being treated with SnO_2_ NP were observed and found that the cells shrank significantly. The cell density decreased, which further demonstrated that SnO_2_ NP could exert cytotoxicity on oral cancer cells.

It is known that the cytotoxicity induced by SnO_2_ NP is involved in the generation of oxidative stress, and Ahamed et al. (2018) demonstrated that in breast cancer cells SnO_2_ NP induced a dose-dependent increase in the production of oxidative products. As we all know, the production and removal of ROS in a normal healthy body are in a dynamic balance. When harmful factors destroy this balance, the level of free radical scavenging enzymes in the body will increase the production of ROS. Besides, it may also trigger the cleavage of lipid peroxides to MDA, which plays a cytotoxic role (Kannan and Jain, 2000). Therefore, MDA and ROS are effective markers of the occurrence of oxidative stress in the body. This study found that SnO2 NPs increased MDA and ROS levels in CAL-27 and SCC-9 cells, and by detecting the expression of oxidative stress-related factors Cytochrome C, it was found that SnO2 NPs can induce oral cancer cells to produce an increased amount of Cytochrome C, thereby increasing oxidative stress. The above results indicated that SnO_2_ NP could cause cell damage by inducing oxidative stress.

Cytochrome c plays a vital role in apoptosis in addition to its role in oxidative stress. It is known that caspase-3 and caspase-9 belong to the cysteine aspartic protease family and act as intermediary proteins in the degradation of proteolytic enzymes during apoptosis. In mammals, cytochrome c together with apoptosis-activating factor 1 activates caspase 9, which subsequently leads to caspase-3 activation, finally triggering a cascading waterfall effect and inducing apoptosis. This study found that SnO2 NPs can significantly increase the number of apoptosis of oral cancer cells, and the expression levels of apoptosis-related proteins Cleared Caspase-3 and Cleared Caspase-3 were also increased at different levels. These results suggest that SnO_2_ NP can cause cell damage by inducing apoptosis.

In addition to apoptosis and oxidative stress, the effects of SnO_2_ NP treatment on migration and invasion of oral cancer cells were also examined in this study. The results showed that oral cancer cells exposed to SnO_2_ NP can significantly reduce their migration and invasion ability, and this inhibitory effect became more apparent, with the prolonged exposure time. In addition to visually observing changes in cell function, the mRNA and protein expression of migration and invasion related genes MMP-2 and MMP-9 were also detected. MMPs are known to be essential for tumor angiogenesis and metastasis, and the destruction of the basement membrane by activating MMPs is a crucial step for cancer invasion and metastasis (Lin et al., 2014). Therefore, inhibition of MMPs expression can provide an initial target for preventing tumor metastasis. This study found that after treatment with SnO_2_ NP, both MMP-2 and MMP-9 protein and mRNA expressions in oral cancer cells were down-regulated by varying degrees. This result is consistent with the results of migration and invasion experiments, suggesting that SnO_2_ NP can inhibit the migration and invasion of oral cancer cells.

Despite the results shown in this study, because anti-tumor polymer nano-drugs are a new research hotspot and the research history is still relatively short, there are still a series of problems in this research, such as the lack of in-depth understanding of metabolic kinetics and biodistribution of the nano-drugs. For targeted tumor therapy, key factors (such as particle size, charge, surface chemistry) are still lacking in-depth exploration. Furthermore, due to the complexity of tumors, the therapeutic effect of nanomedicine in different tumor types and tumors with different stages of progressions may be different. Nevertheless, nanotechnology is still a very important hot subject that affects medicine. Although the research and development of nanotechnology-applied medicine are still only in its early stages, due to its rapid development, it will surely develop into a new discipline and new industry.

## Conclusion

In summary, we found in this study that SnO_2_ NP can exert cytotoxic effects on oral cancer cells by inhibiting cell proliferation, migration, and invasion abilities, and can also induce oxidative stress and apoptosis. Therefore, it is indicated that SnO_2_ NP may have anti-oral cancer effects, which can provide a basis for future research and clinical application and has important theoretical value. However, more in-depth animal experiments and clinical trials are still needed to confirm its clinical value. This is a major limitation of this study and a direction for future research.

## Data Availability Statement

The original contributions presented in the study are included in the article/supplementary material, further inquiries can be directed to the corresponding author/s.

## Ethics Statement

The animal study was reviewed and approved by the Animal Care and Use Committee at Jilin University.

## Author Contributions

HY and BZ proposed and designed the experiments. HL and QL carried out the experiments with the help of YL and XS. HL and QL drafted the manuscript and interpreted the data. HL, QL, HY, and BZ revised the manuscript. All authors contributed to the article and approved the submitted version.

## Conflict of Interest

The authors declare that the research was conducted in the absence of any commercial or financial relationships that could be construed as a potential conflict of interest.
